# Expression and Secretion of Human Proinsulin-B10 from Mouse Salivary Glands: Implications for the Treatment of Type I Diabetes Mellitus

**DOI:** 10.1371/journal.pone.0059222

**Published:** 2013-03-15

**Authors:** Anne M. Rowzee, Paola J. Perez-Riveros, Changyu Zheng, Sarah Krygowski, Bruce J. Baum, Niamh X. Cawley

**Affiliations:** 1 Molecular Physiology and Therapeutics Branch, National Institute of Dental and Craniofacial Research, National Institutes of Health, Bethesda, Maryland, United States of America; 2 Section on Cellular Neurobiology, Eunice Kennedy Shriver National Institute of Child Health and Human Development, National Institutes of Health, Bethesda, Maryland, United States of America; National Institute of Dental and Craniofacial Research, United States of America

## Abstract

Adenovirus (Ad) mediated expression of therapeutic proteins from salivary glands can result in the delivery of biologically active proteins into the circulation where they impart their physiological function. In recent years, Ad vector delivery to salivary glands (SGs) has emerged as a viable option for gene therapy. Here, we engineered a variant of human proinsulin (hProinsulin-B10) into an Ad vector and demonstrated its ability to transduce cell lines, and express a bioactive protein that induces the phosphorylation of AKT, a key insulin signaling molecule. We also examined its expression in mice following delivery of the vector to the parotid gland (PTG), the submandibular gland (SMG) or to the liver via the tail vein and assessed transgenic protein expression and vector containment for each delivery method. In all cases, hProinsulin-B10 was expressed and secreted into the circulation. Lower levels of circulating hProinsulin-B10 were obtained from the PTG while higher levels were obtained from the tail vein and the SMG; however, vector particle containment was best when delivered to the SMG. Expression of hProinsulin-B10 in the SMG of chemically induced diabetic mice prevented excessive hyperglycemia observed in untreated mice. These results demonstrate that hProinsulin-B10 can be expressed and secreted into the circulation from SGs and can function physiologically *in vivo*. The ability to remediate a diabetic phenotype in a model of type 1 diabetes mellitus is the first step in an effort that may lead to a possible therapy for diabetes.

## Introduction

It has been demonstrated previously that salivary glands (SGs) are a safe and relatively straightforward target for gene transfer in several animal models and humans [1], [2], [3]. A Phase I clinical study to evaluate the safety of gene transfer to SGs in humans is currently in progress (reviewed in Baum et al., 2010) [4]. The SG provides an attractive alternative to many previously employed gene therapy target organs; SGs are not critical for life organs and a single SG can be removed in the event of a severe adverse reaction with relatively low morbidity [1]. Furthermore, human SGs are well-encapsulated, which prevents the spread of viral vectors beyond the target tissue and thus reduces the likelihood of systemic adverse events [5]. A minimally invasive technique is used to deliver vector via the main excretory duct of the SG and the luminal membrane of nearly every salivary epithelial cell is in contact with this duct. This characteristic is advantageous because a small and undiluted volume of vector suspension has the potential to transduce nearly every epithelial cell within the targeted gland. SG epithelial cells can produce and secrete large amounts of protein primarily into the saliva via the exocrine, regulated secretory pathway (RSP). SG cells can also secrete protein into the circulation via their basolateral membranes, which is thought to occur via the constitutive secretory pathway (CSP) [6], [7]. Although the route of secretion for transgenic proteins is not entirely predictable *in vivo*, we have shown that vectors targeted to the SG can lead to secretion of therapeutic levels of many transgenic proteins, such as growth hormone [8], parathyroid hormone [9], erythropoietin [10] and glucagon-like peptide-1 (GLP-1) [2] via the CSP as a potential treatment for endocrine disorders.

Diabetes mellitus (DM) is an endocrine disorder that affects 8.3% of the U.S. population and is a causative factor for other chronic health problems including heart disease, blindness and kidney failure. Insulin-dependent DM, or type I DM, results from the destruction of pancreatic β-cells where insulin is normally made. Current therapies to treat DM rely on systemic replacement of insulin typically requiring multiple daily injections that are expensive, inconvenient and uncomfortable. Although exogenous insulin therapy improves glycemic control, it carries the risk of hypoglycemia and can be cost-prohibitive. As such, current treatment methods are merely tolerated and alternative methods of protein production and delivery are being explored and utilized. Gene therapeutic approaches for ectopic expression of the pre-proinsulin gene or a variation thereof have proven to be an effective alternative to insulin injections in animal models [11], [12], [13], [14], [15], [16]. Insulin production following gene delivery to liver [17] has been demonstrated to reduce blood glucose levels. However, clinical application of this strategy is limited because the procedures to target this organ are invasive and, severe patient morbidity is likely in the event of an adverse reaction [18].

To assess the feasibility of SG gene therapy for treatment of DM, we generated a recombinant adenoviral (rAd) serotype 5 vector that encodes the cDNA for wild type human proinsulin (hProinsulin-WT) under the direction of the cytomegalovirus (CMV) promoter. We also generated a vector encoding the hProinsulin-B10 variant which differs from WT by a single base change that results in a histidine to aspartic acid substitution (His-B10-Asp) in the proinsulin B-chain peptide [19]. In contrast to WT proinsulin that is targeted to the RSP, B10 proinsulin rapidly exits pancreatic β-cells *in vivo* and neuro-endocrine cells *in vitro* via the CSP [20], [21]. This is particularly important considering that secretion via the CSP from polarized SG cells leads to the circulation. The use of proinsulin cDNA rather than mature, processed insulin is also advantageous because serum proinsulin has a longer half-life, yet roughly 25% of the efficacy of mature human insulin, which may reduce the potential for hypoglycemia in the event of chronic overexpression [22], [23].

To establish the functionality of our constructs we first tested the ability of these rAd vectors to transduce cells and secrete human insulin (hInsulin) or hProinsulin *in vitro.* We also evaluated the bioactivity of secreted transgenic hProinsulin-WT and hProinsulin-B10 versus recombinant human insulin (rhInsulin) *in vitro* to demonstrate that the expressed proteins could function in insulin signaling. The rAd-hProinsulin-B10 vector was subsequently delivered *in vivo* via three routes; tail vein injection, and PTG or SMG instillation. Thereafter, transgenic protein levels were assessed in the circulation and the ability of the transgenic protein to lower blood glucose tested in non-diabetic mice. Finally, the rAd-hProinsulin-B10 vector was delivered to mice with chemically-induced diabetes via the SMGs to assess its ability to reduce hyperglycemia in a mouse model system.

## Materials and Methods

### Ethics Statement

All animal studies described herein were done with the approval of the Animal Care and Use Committee, NIDCR, NIH. To reduce pain and distress of the animals, they were anesthetized with ketamine:xylazine prior to viral instillation and were monitored regularly until recovery. For most experiments, euthanasia was carried out first by anesthesia with ketamine:xylazine so that saliva could be collected and then by cervical dislocation. Animals from the final experiment were euthanized by CO_2_ inhalation.

### Construction of Plasmids and Recombinant Adenoviral Vectors

The human proinsulin-B10 variant cDNA was PCR amplified from a hProinsulin-B10 plasmid generated by Lofstrand laboratories (Gaithersburg, MD) using Platinum *Taq* DNA Polymerase High Fidelity (Invitrogen, Carlsbad, CA) and sense and antisense primers designed to add the SalI and HindIII restriction enzyme sites, respectively. Primer sequences were as follows: sense - 5′-GCCATATAGTCGACGGAATTCTGCCATGGCCCTGTGGATGCGCC-3′ and antisense 5′-CGTACGTGCAAGCTTGCTGGTTCAAGGGCTTTATTCCATCTCTC-3′. Ten microliters of the final PCR product were purified using the QIAquick PCR purification kit (Qiagen, Valencia, CA) and the purified hProinsulin-B10 product was inserted into the SalI/HindIII restriction enzyme site of the adenoviral shuttle vector, pACCMV [24]. The pAC-proinsulin-wild type (WT) vector was generated by site directed mutagenesis of the pAC-proinsulin-B10 vector using the QuikChange II XL Site-Directed Mutagenesis Kit (Stratagene, La Jolla, CA) and the following mutation primers: sense 5′-CCTGTGCGGCTCACACCTGGTGGAAGC-3′ and antisense 5′-GCTTCCACCAGGTGTGAGCCGCACAGG-3′. After sequence confirmation of pAC-proinsulin-B10 and pAC-proinsulin-WT, both shuttle vectors were used to generate, purify and titer rAd vectors as detailed in Zheng and Baum, 2005 [24].

### Cell Culture

All cells were maintained at 37°C in humidified 5% CO_2_. Rat A5 [25] cells were maintained as in Zheng and Baum, 2005 [24]. Mouse NIT1 cells [26] were maintained in 1X high glucose DMEM (4.5 g/L D-glucose) plus 10% heat-inactivated fetal bovine serum (FBS), 100 U/ml Penicillin, 100 ug/ml streptomycin and 2 mM glutamine all from Gibco/Invitrogen. HEK293 cells were maintained in modified IMEM (Gibco) plus supplements as listed for NIT1 cells.

### *In vitro* Transduction

A5 and NIT1 cells were seeded on 12-well tissue culture plates and allowed to attach. The following day, the cells in one well of each plate were counted and the volume of vector stock required to infect at doses of 1, 10, 100 and 1000 viral particles/cell was calculated. Media was replaced in all remaining wells and n = 2 wells for each vector dose and for control (0 vector particles/cell) were incubated overnight. Media from each well were then collected independently, cleared of cells and stored at −80°C until ELISA analysis. HEK293 cells were seeded on 6-well tissue culture plates, allowed to attach, counted, and n = 3 wells per vector dose and control were transduced and samples were collected as for A5 and NIT1 cells. To generate conditioned media (CM) for *in vitro* bioactivity assays, confluent 150 mm dishes of HEK293 cells were switched to serum-free medium (SFM) and incubated with no vector (Control CM) or with either rAd-proinsulin-WT or rAd-proinsulin-B10. The following day, CM were collected, cleared of cells, and stored at −80°C. Most samples were assayed by ELISA for expression of human insulin and human proinsulin.

### *In vitro* hProinsulin Bioactivity

HEK293 cells were seeded in 6-well tissue culture dishes at a density of 4×10^5^ cells/well and allowed to attach for 48 h. Cells were then switched to SFM for 2 h to decrease signal transduction then 3 wells/group were treated with Control CM (no vector), 4.5 nM WT hProinsulin CM from rAd-proinsulin-WT-transduced cells, 4.5 nM B10 hProinsulin CM from rAd-proinsulin-B10-transduced cells or 4.5 nM recombinant human insulin (rhInsulin) diluted in SFM (Sigma). Fifteen minutes later, treatment was stopped using ice-cold 1X PBS and cell pellets were collected. Cell lysates were prepared by incubating pellets with 1X Cell Lysis Buffer (Cell Signaling, Beverly, MA) plus 1 mM phenyl methyl sulphonyl fluoride (PMSF) (Sigma) for 30 min on ice with periodic vortexing followed by centrifugation in a microcentrifuge at 10,000 RPM, for 5 min at 4°C. Cleared lysates were transferred to fresh tubes and protein concentration was determined using the DC Protein Assay (modified Lowry Assay, BioRad, Hercules, CA).

### Western Immunoblot Analysis

Twenty micrograms of total protein from *in vitro* bioactivity experiment samples were subjected to denaturing SDS-PAGE using a 4–12% Bis-Tris gradient gel (Invitrogen), following by western immunoblotting onto a nitrocellulose membrane (Invitrogen). The membrane was blocked with 5% milk in 1X TBS-0.1% Tween (TBS-T) prior to incubation with Phospho-Akt (Ser473) primary antibody (Cell Signaling) diluted 1∶500 in 5% BSA in 1X TBS-T. The membrane was then washed with 1X TBS-T prior to incubation with Cy3 conjugated, goat-anti-rabbit secondary antibody (Amersham/GE Healthcare, Piscataway, NJ) diluted 1∶2500 in 5% milk/1X TBS-T and visualization using the Typhoon 9410 Variable Mode Imager (Amersham). The membrane was then stripped of previous antibodies by incubation with 60 mM Tris-HCl, pH 6.8, 2% SDS and 0.7% beta-mercaptoethanol at 50°C in a shaking water bath for 30 min. Membranes were then washed with 1X TBS, and blocked and incubated with total Akt primary antibody (Cell Signaling) using the same conditions as listed above. The membrane was then washed with 1X TBS-T prior to incubation with a Cy5-conjugated, goat-anti-rabbit secondary and visualization as described above. Band signal intensities were measured using Scion Image Software (Scion Corporation).

### *In vivo* Transduction

For all *in vivo* experiments, 8–10 week old, male, Balb/c mice were used and either sterile saline (Control) or rAd-proinsulin-B10 particles diluted in sterile saline were delivered. For I.V. delivery of vector, 100 uL of vector suspension or saline alone were injected into the tail vein. For PTG delivery, both glands were cannulated and 25 uL of rAd-proinsulin-B10 particles or saline alone were delivered by retroductal infusion as described in [27]. For SMG delivery, both glands were cannulated and 50 uL of rAd-proinsulin-B10 particles or saline alone were delivered by retroductal infusion as described in Voutetakis, A et al 2010 [2].

### *In vivo* Sample Collection

At 24 h and 48 h post-vector delivery, whole blood samples were obtained by retro-orbital bleeding. A drop of each sample was applied to a hand-held glucose meter (OneTouch Ultra, LifeScan, Inc., Milpitas, CA) to determine blood glucose levels and the remaining sample was allowed to clot at 4°C overnight, centrifuged at 13000 RPM for 10 min and serum was transferred to a fresh tube and stored at −80°C until analysis. Following blood sample collection at 48 h post-transduction, animals were anesthetized and saliva was collected as in Voutetakis, A et al 2010 [2]. Animals were sacrificed after saliva collection and liver, spleen and salivary gland tissues were collected, snap frozen in liquid NO_2_ and stored at −80°C.

### ELISA Analysis of Human Insulin and Human Proinsulin

All measurements of hInsulin were made in duplicate with the Ultrasensitive Insulin ELISA kit (Mercodia, Sweden) and data were converted from mU/L to pM using a factor of 6 [28]. All measurements of hProinsulin were made in duplicate with the Intact Human Proinsulin ELISA Kit (Millipore, Billerica, MA). Liver, spleen and SG tissue protein extracts were prepared by homogenizing samples in 1X Cell Lysis Buffer (Cell Signaling) with 1 mM PMSF followed by sample processing and total protein quantification as described above for *in vitro* experiments. Fifteen micrograms of liver, spleen and parotid protein and 30 µg of SMG protein were used per well for each ELISA.

### DNA Extraction and QPCR Analysis of Vector Copy Number *in vivo*

Ten milligrams of all tissue samples were lysed to extract genomic DNA using the DNeasy Blood and Tissue kit, Animal Tissue Spin-column Protocol with all optional steps performed (Qiagen, Valencia, CA). In experiments where 2 SGs were transduced, 5 mg pieces of each gland were combined and DNA was then isolated. Vector copies present in each tissue sample were measured using absolute quantitation QPCR against a dilution series of the pACCMV shuttle vector to serve as a standard curve. All standards and samples were run in triplicate on the Applied Biosystems StepOne Plus real-time QPCR system (Applied Biosystems, Foster City, CA) and data analysis was performed with the associated StepOne Software v2.1. Each reaction contained 100 ng of genomic DNA, 2X SYBR green PCR master mix (Applied Biosystems), 7.5 pmole of E2q1 primer: 5′-GCAGAACCACCAGCACAGTGT-3′, and 7.5 pmole of E2q2 primer: 5′-TCCACGCATTTCCTTCTAAGCTA-3′. Reaction conditions were as follows: 95°C for 2 min, 95°C for 10 min, followed by 40 cycles of 95°C for 15 sec and 60°C for 1 min.

### ALX-Diabetes

Mice were fasted for 16 h prior to intraperitoneal (IP) injection of 200****mg/kg Alloxan monohydrate (ALX, Sigma) after which they were fed ad libitum. Four hours following ALX injection, mice were given an IP injection of 3 gm/kg dextrose (Sigma) to prevent severe hypoglycemia induced by ALX. Beginning the following morning, blood glucose for all mice was measured daily using a drop of blood obtained via tail nick and a handheld glucose meter. When blood glucose values reached >200 mg/dl, mice were given 1 U of HumulinN (Lilly, Indianapolis, IN) by subcutaneous injection twice daily (morning and evening) for 5 days. On the morning of the sixth day, HumulinN treatment was discontinued and both SMGs were transduced with either 5×10^9^ particles of rAd-proinsulin-B10 or rAd-Control (empty vector). Twenty four hours later, whole blood was collected in micro tubes containing a Serum-Gel clotting activator (Sarstedt AG & Co., Numbrecht, Germany), centrifuged at 10,000×g for 5 m and serum was transferred to fresh tubes and stored at −80°C until analysis. Serum glucose levels were measured as above with a handheld glucose meter.

### Statistics

Statistical analyses were performed using SPSS Software 15.0 for Windows (LEAD Technologies, Inc.) as indicated in figure legends.

## Results

### Human Insulin and Proinsulin Secretion following *in vitro* Transduction with rAd-proinsulin-WT or rAd-proinsulin-B10

**Figure 1:**
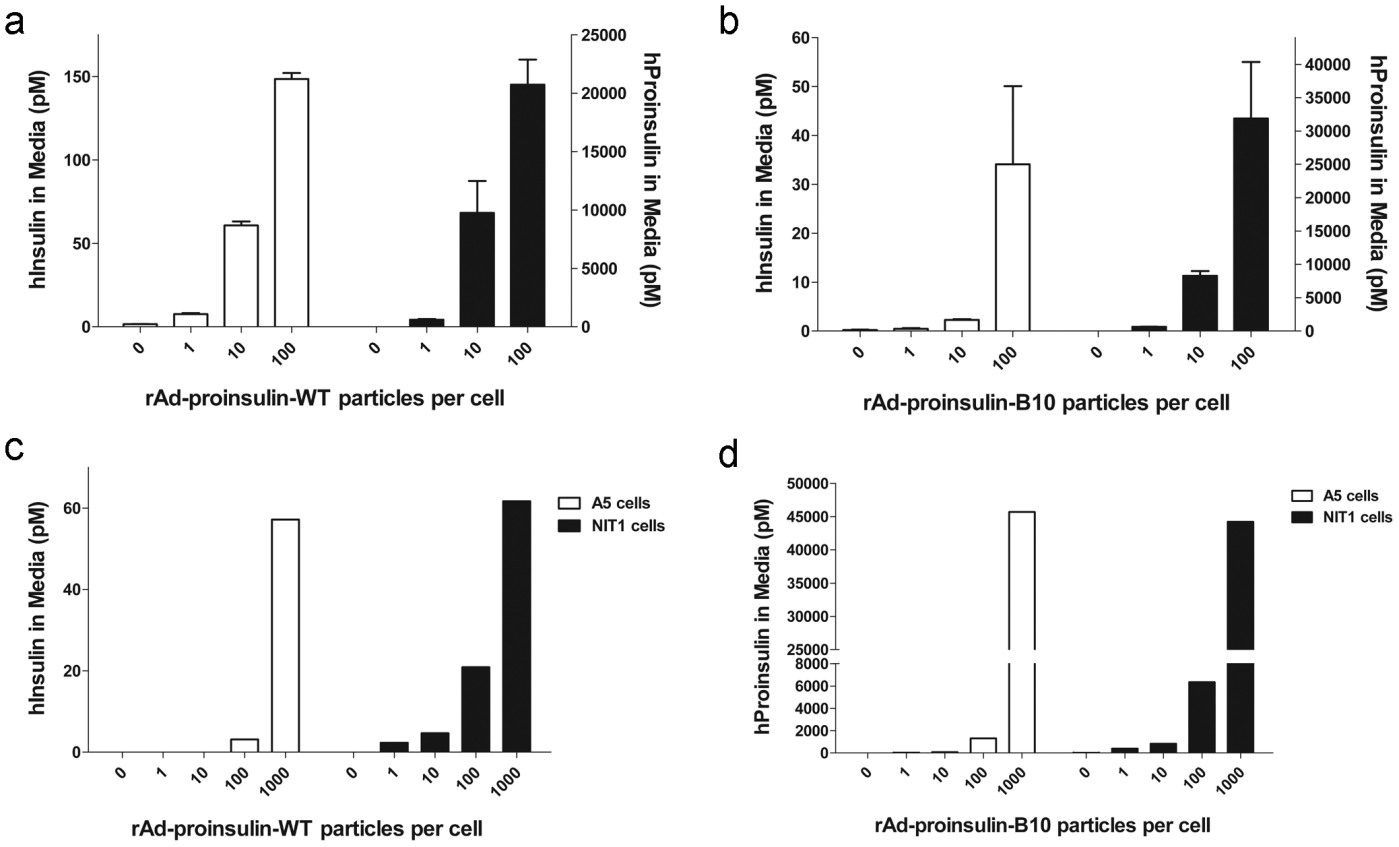
Human insulin and proinsulin secretion following *in vitro* transduction with rAd-proinsulin-WT or rAd-proinsulin-B10. (**a, b**) HEK293 cells were transduced with rAd-proinsulin-WT (**a**) or –B10 (**b**) as indicated and media samples were assayed 24 hr later for both hInsulin and hProinsulin expression. Bars indicate mean+SEM concentration of hInsulin (open bars, left axes) or of hProinsulin (filled bars, right axes) for n = 3 samples per group. (**c,d**) Rat A5 salivary gland cells and mouse NIT1 pancreatic beta-cells were transduced with rAd-proinsulin-WT (**c**) or –B10 (**d**) particles at the doses indicated. Media were collected 24 h later and insulin species were quantified by ELISAs. Bars indicate mean concentration of hInsulin (**c**) or hProinsulin (**d**) in the media of n = 2 samples.

Human epithelial cells (293 cells) transduced with increasing doses of rAd-proinsulin-WT or –B10 displayed dose-dependent secretion of both human insulin (hInsulin) and hProinsulin into culture media (Fig. 1 a and b, respectively). As expected, cells transduced with the WT vector secreted more mature insulin than those transduced with B10 vector whereas cells transduced with the B10 vector secreted higher levels of hProinsulin than WT vector-transduced cells. The 293 cells transduced with WT vector secreted over 100X more hProinsulin than hInsulin (Fig. 1a, compare open bars with filled bars). This is likely due to the low expression of insulin prohormone processing enzymes and the lack of an RSP in these cells where proinsulin processing typically occurs. As such, the majority of the hProinsulin expressed in 293 cells is rapidly secreted without post-translational enzymatic processing and prohormone maturation.

Both vectors were also evaluated in rat A5 SMG cells and mouse NIT1 pancreatic β-cells *in vitro* to determine the ability of salivary gland cells and pancreatic β-cells to express and secrete human insulin proteins, and to ensure that hProinsulin-B10 expressed from our recombinant vectors could be secreted via a CSP. For both rat A5 SMG and mouse NIT1 cells transduced with the WT vector, the amounts of secreted hInsulin (Fig. 1c, A5 cells, open bars; NIT1 cells, filled bars) were slightly less but comparable to those observed for the 293 cells. Similarly, for both cell types transduced with the B10 vector, the levels of secreted hProinsulin (Fig. 1d, A5 cells, open bars; NIT1 cells, filled bars) were also comparable to the 293 cells. NIT1 cells have both a CSP and an intact RSP that can be stimulated to secrete murine insulin [29]. The studies shown here were performed under non-stimulating conditions and demonstrate that NIT1 cells can secrete hProinsulin (Fig. 1d, filled bars) via their CSP in a dose dependent fashion despite having a RSP. It should be noted that neither hInsulin nor hProinsulin were detected in non-transduced NIT1 mouse cells, verifying that the ELISAs used in these studies are species-specific and will not cross-react with endogenous mouse insulin molecules in the course of *in vivo* experiments.

### Human Proinsulin-WT and hProinsulin-B10 expressed by Transduced HEK293 Cells is Bioactive *in vitro*

**Figure 2:**
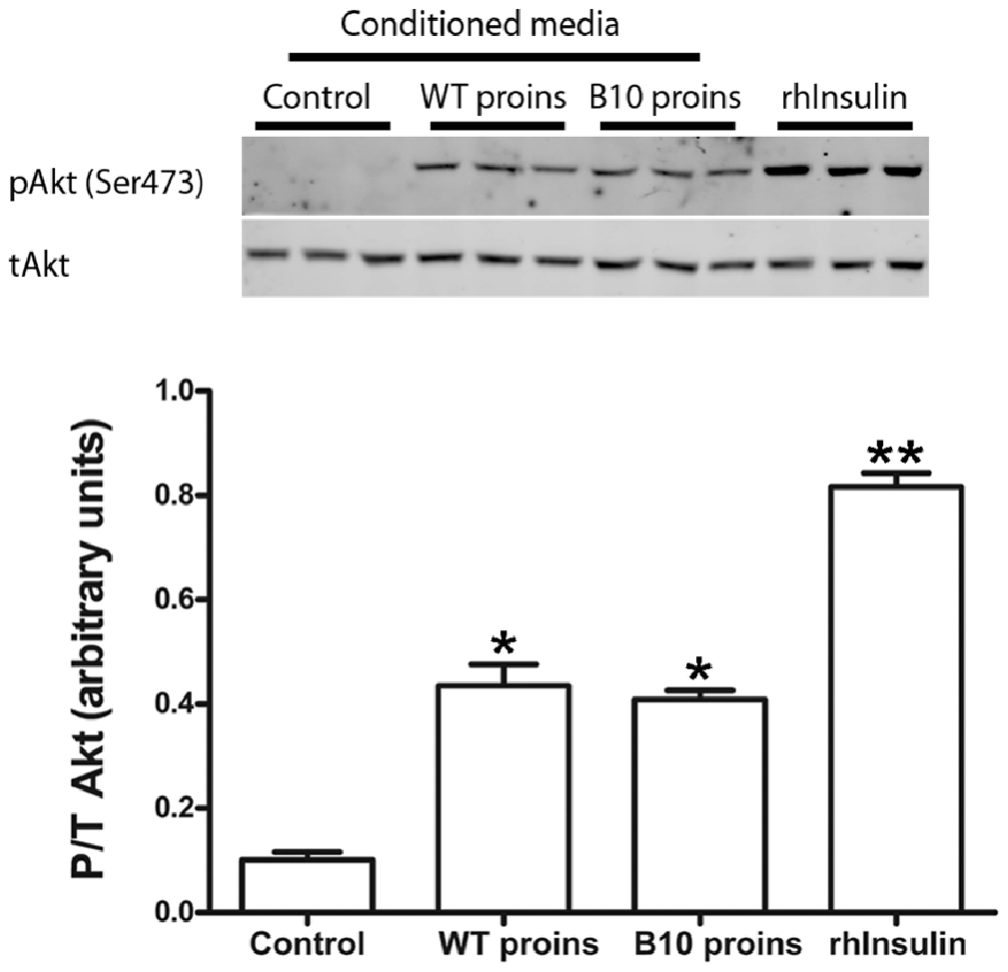
Human Proinsulin-WT and Proinsulin-B10 expressed by transduced HEK293 cells is bioactive *in vitro*. Confluent HEK293 cells were switched to serum-free medium with or without rAd-proinsulin-WT or rAd-proinsulin-B10; 24 h later, conditioned media (CM) were collected and hProinsulin concentration was determined by ELISA. Fresh HEK293 cells were seeded, serum starved for 2 h, and treated for 15 min with Control CM (no vector), 10 nM hProinsulin-WT CM (WT proins), 10 nM hProinsulin-B10 CM (B10 proins) or 10 nM recombinant human insulin (rhInsulin). Protein lysates were collected and analyzed by western immunoblot for phospho-Akt(Ser473) and total Akt. Immunoblot band intensities were measured and bars indicate mean+SEM of the P/T Akt ratio for n = 3 samples; *, P<0.0001 vs. control, **, P<0.0001 vs. all (ANOVA).

We determined that serum-starved 293 cells respond in a dose-dependent manner to rhInsulin treatment by phosphorylation of Akt on Serine 473 (Fig. S1). Therefore, using Akt_Ser473_ as a biological readout of insulin receptor (InsR) signaling activity [30], [31], we tested the ability of hProinsulin-WT and hProinsulin-B10 expressed *in vitro* to stimulate InsR signaling in serum-starved 293 cells. Figure 2 demonstrates that conditioned media containing either 10 nM hProinsulin-WT or 10 nM hProinsulin-B10 significantly stimulated InsR signaling compared to controls, with approximately 40% efficacy compared to rhInsulin *in vitro*. The small amount of mature insulin in the conditioned media (shown in figure 1) was likely to only marginally contribute to InsR signaling in this assay, if at all.

### Dose Dependent Secretion of hProinsulin in Mice Transduced with rAd-proinsulin-B10 via I.V. Injection

**Figure 3:**
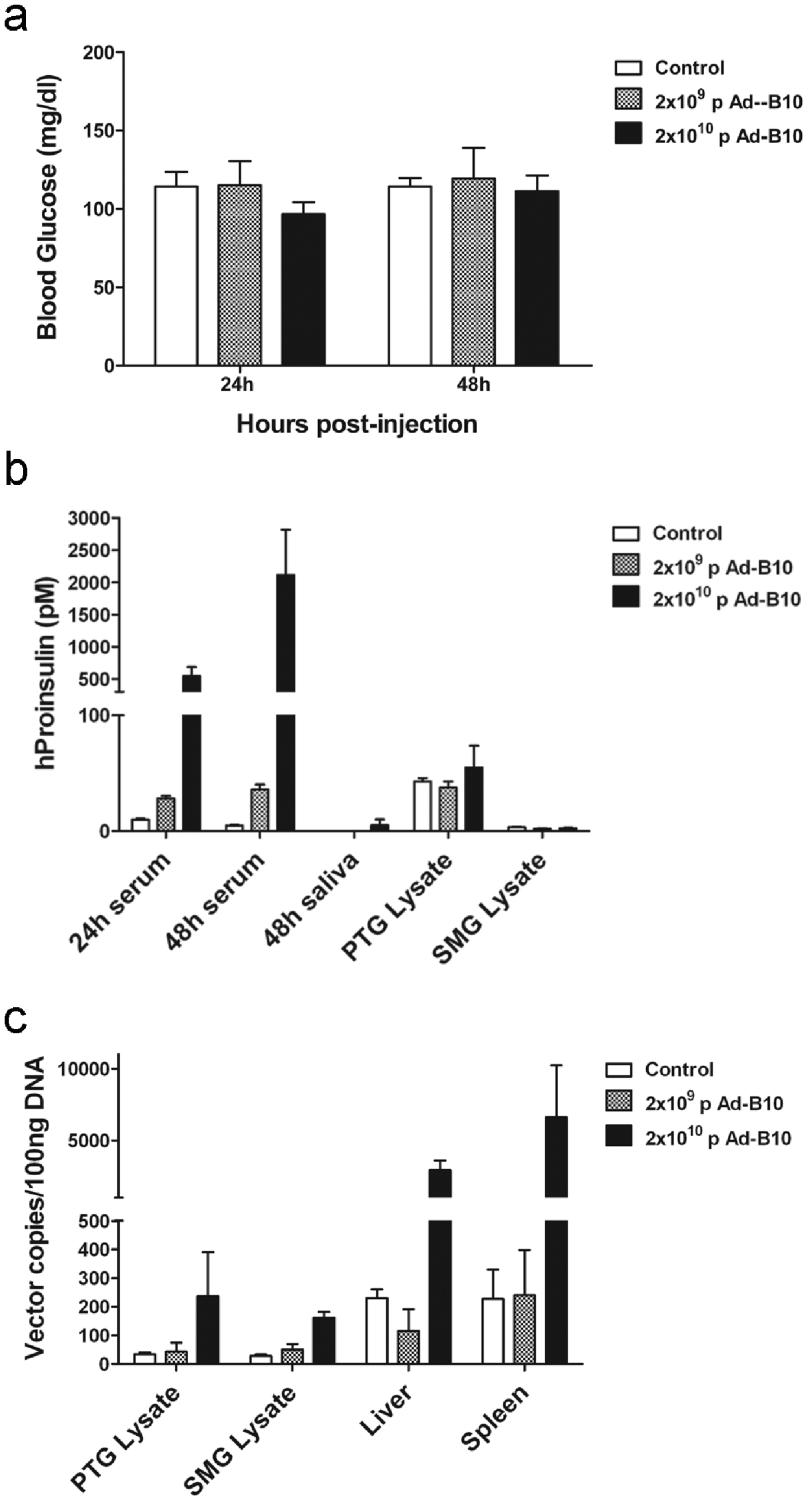
Dose dependent secretion of hProinsulin in non-diabetic mice transduced with rAd-proinsulin-B10 via I.V. injection. rAd-proinsulin-B10 particles or saline (Control) was delivered by I.V. injection. Serum samples were obtained at 24 h and 48 h post-transduction and saliva, parotid gland (PTG), submandibular gland (SMG), liver and spleen tissues were collected at the conclusion of the experiment (48 h). (**a**) Blood glucose (mg/dl) measurements at 24 h and 48 h post-transduction. No statistical difference in glucose levels was observed between groups (ANOVA) (**b**) Sera, saliva and protein lysates from PTG and SMG were assayed for hProinsulin (pM) by ELISA. (**c**) QPCR analysis of rAd vector genome tissue distribution at the conclusion of the experiment. Data are expressed as rAd genome copies detected in 100 ng of tissue genomic DNA. For all panels, bars indicate mean+SEM of n = 3 mice per group.

The observations that expression and secretion of insulin species were higher following transduction of cells with the B10 vector and that secreted hProinsulin-B10 was equally as effective as hProinsulin-WT at stimulating the InsR *in vitro*, led us to continue *in vivo* experiments using the rAd-proinsulin-B10 vector solely. Intravenous (I.V.) injection of this vector was first performed to target it to the liver in order to achieve high levels of transgenic protein secretion into the circulation and to determine if hProinsulin in circulation would alter glucose homeostasis in non-diabetic mice. Non-fasting blood glucose levels of mice transduced with rAd-proinsulin-B10 remained unchanged from controls at 24 h and 48 h post-injection (Fig. 3a) even though elevated levels of hProinsulin were detected in the serum of mice treated with the highest vector dose at these times (Fig. 3b). Saliva and salivary gland tissues were also analyzed for the presence of hProinsulin at 48 h post-injection (Fig. 3b) to quantify background levels of hProinsulin in these compartments and provide a basis of comparison for subsequent *in vivo* salivary gland delivery experiments. In the event that hProinsulin-B10 produced by the liver was being processed to mature hInsulin-B10 prior to secretion, serum samples were also analyzed for the presence of hInsulin specifically and found to be well below 6 pM, the detection limit of the ELISA kit. QPCR analysis of DNA from several tissues for the presence of vector genomes verified that the I.V. injections resulted in delivery of the rAd-proinsulin-B10 vector to the liver and also to spleen, but with some dissemination to SGs at the highest vector dose (∼10%; Fig. 3c). Taken together these data indicate that hProinsulin-B10 expressed from liver and spleen is being trafficked and constitutively secreted *in vivo* as predicted by our *in vitro* experiments described above.

### rAd-proinsulin-B10 Transduced Parotid Glands of Non-diabetic Mice Secrete hProinsulin to the Circulation and Restrict Extra-glandular Vector Dissemination

**Figure 4:**
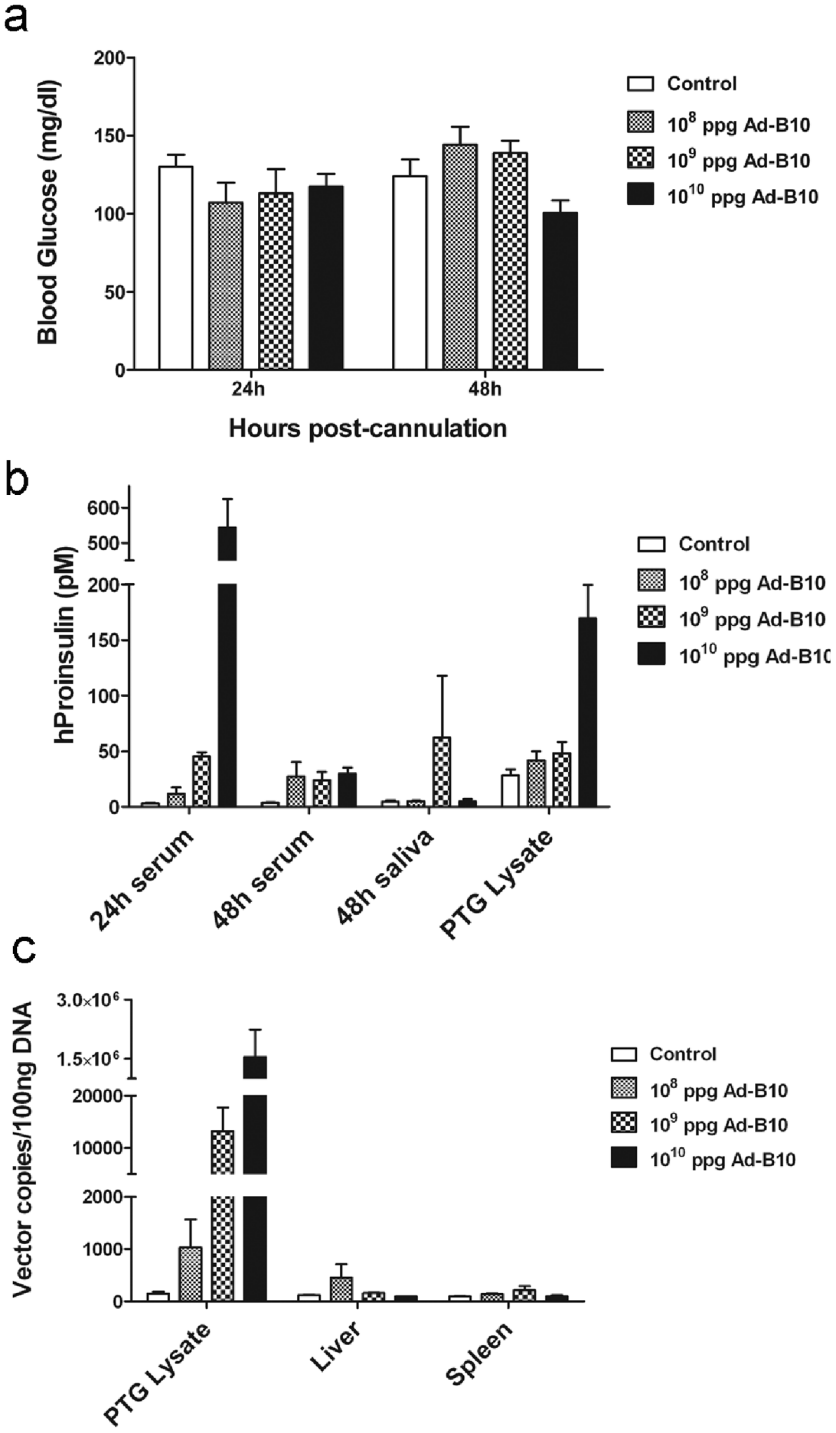
rAd-proinsulin-B10 transduced PTGs secrete hProinsulin to the circulation and restrict extra-glandular vector dissemination. rAd-proinsulin-B10 or saline (Control) was delivered to both PTGs of non-diabetic mice by retroductal infusion. Blood samples were obtained at 24 h and 48 h post-transduction and saliva and PTG samples were collected at the conclusion of the experiment (48 h). (**a**) Blood glucose (mg/dl) measurements at 24 h and 48 h post-transduction. No statistical difference in glucose levels was observed between groups (ANOVA) (**b**) Sera, saliva and PTG protein lysates were assayed for hProinsulin (pM) by ELISA. (**c**) QPCR analysis of rAd vector genome distribution in tissues at the conclusion of the experiment. Data are expressed as rAd genome copies detected in 100 ng of genomic DNA. For all panels, ppg = particles per gland and bars indicate mean+SEM of n = 4 mice for 10^9^ ppg and Control groups, and n = 5 for all other groups.

The rAd-proinsulin-B10 vector was next delivered in increasing doses to the PTGs via cannulation and retroductal infusion to assess the secretion route of hProinsulin-B10 from PTG cells *in vivo* and to verify that this method of vector delivery did not result in significant spread of the viral vector outside of this gland. Delivery of the B10 vector to PTGs did not significantly reduce blood glucose levels in non-diabetic mice at either 24 h or 48 h post-cannulation (Fig. 4a). In contrast with I.V. delivery where hProinsulin was present in circulation at 24 h and increased through 48 h, circulating hProinsulin following PTG delivery was highest at 24 h with a decrease at 48 h (Fig. 4b). As anticipated by the markedly smaller size of the PTG, peak levels of hProinsulin secretion from PTGs was roughly 5× lower than that from liver (compare Fig. 3b and 4b).

Sera, saliva and PTG tissue samples collected at the end of the experiment (48 h post-cannulation) were also analyzed for hProinsulin to determine the distribution of transgenic protein (Fig. 4b). Although serum hProinsulin dramatically decreased in mice transduced with 10^10^ particles per gland (ppg) by 48 h post-cannulation, hProinsulin was detected above background (Control) in all serum samples at this time. Excepting a single outlier in the 10^9^ ppg group, hProinsulin was not detected in the saliva of rAd-proinsulin-B10 treated mice nor above background in the PTG lysates of mice treated with 10^8^ or 10^9^ ppg. Human proinsulin was detected in the PTG lysates of mice treated with 10^10^ ppg, perhaps indicating that the transgenic protein is not being efficiently secreted at this high vector dose.

To verify that the hProinsulin detected in serum samples was due solely to transduction of PTG cells and the subsequent expression and secretion of the transgenic protein, we analyzed liver and spleen samples for the presence of vector genomic DNA. As shown before in Figure 3c, vector injected into the tail vein transduces liver and spleen tissues. If PTG cannulation and vector delivery resulted in spread of the rAd-proinsulin-B10 vector to the blood stream, vector genome copies would be detected in liver or spleen tissues. Figure 4c shows that at 48 h post-cannulation of the PTGs the majority of viral DNA was detected in PTG lysates at all vector doses, and at the highest dose no vector DNA was detected in liver or spleen tissues when compared to that in non-transduced Control samples.

### Dose Dependent Endocrine Secretion of hProinsulin and Subsequent Decreases in Blood Glucose Levels in Non-diabetic Mice Treated with rAd-proinsulin-B10 via SMG Transduction

**Figure 5:**
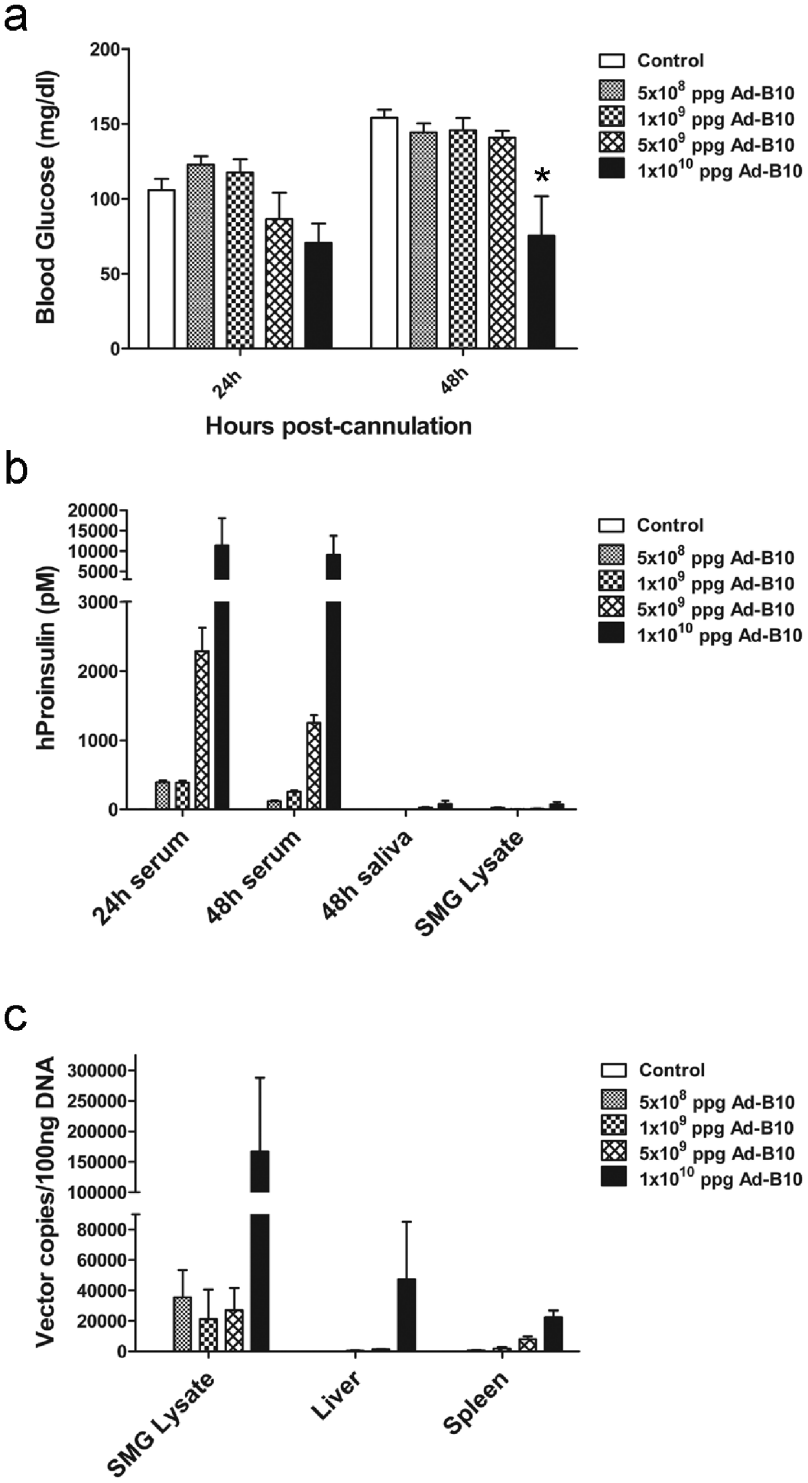
Dose dependent endocrine secretion of hProinsulin and blood glucose levels in mice treated with rAd-proinsulin-B10 via SMG transduction. rAd-proinsulin-B10 or saline (Control) was delivered to both SMG by retroductal infusion. Serum samples were obtained at 24 h and 48 h post-transduction and saliva and SMG samples were collected at the conclusion of the experiment (48 h). (**a**) Blood glucose (mg/dl) measurements at 24 h and 48 h post-transduction. (**b**) Sera, saliva and SMG protein lysates were assayed for hProinsulin (pM) by ELISA. (**c**) QPCR analysis of rAd vector genome tissue distribution at the conclusion of the experiment. Data are expressed as rAd genome copies detected in 100 ng of genomic DNA. For all panels, ppg = particles per gland and bars indicate mean+SEM of n = 4; *, P≤0.021 vs. all at 48 h (ANOVA).

In both of the preceding experiments, a high vector dose, 2×10^10^ particles per animal, was required to observe hProinsulin in serum. To determine the efficacy of slightly lower vector doses in SMGs, which typically produce more protein than rodent PTGs [32], dose titration experiments targeting the SMGs were repeated in non-diabetic mice. Transduction of both SMGs with increasing doses of rAd-proinsulin-B10 resulted in a dose-dependent decrease in blood glucose levels at 24 h post-cannulation with a significant decrease in the 10^10^ ppg group compared to controls at 48 h (Fig. 5a). These data correspond with serum hProinsulin data that demonstrate high levels of hProinsulin at 24 h and 48 h post-cannulation in the two highest groups (Fig. 5b). Consistent with previous reports [32], hProinsulin levels in mice receiving 10^10^ ppg via SMG cannulation greatly surpassed those of mice receiving the same number of vector particles via PTG delivery (compare Fig. 4b with Fig. 5b).

Similar to the previous dose titration experiment targeting the PTG, little to no hProinsulin was detected in the saliva or SMG lysates of mice transduced with the B10 vector by SMG cannulation (Fig. 5b). Analysis of genomic DNA from SMG, liver and spleen lysates showed that vectors were primarily contained within SMGs following cannulation and retroductal infusion, except at the highest dose (Fig. 5c). There was large variability in the liver samples from the 10^10^ ppg group, and 2 samples demonstrated levels of vector DNA similar to that of SMG lysates in the lower dose groups. This variability may be due to a technical error during vector delivery, or may indicate that such an elevated vector dose may be unsuitable for SMGs and result in undesirable vector spread.

### Endocrine Secretion of hProinsulin from SMGs Transduced with rAd-proinsulin-B10 Significantly Reduces Serum Glucose in ALX-diabetic Mice

After observing the high levels of serum hProinsulin that were achieved following SMG transduction at 5×10^9^ ppg and the effects on blood glucose levels in non-diabetic mice, we chose to test the ability of rAd-proinsulin-B10 delivery to SMGs as a potential treatment for type I DM. In these experiments, mice were made diabetic with the β-cell toxin Alloxan, and then received twice daily insulin injections to help maintain glucose homeostasis until vector testing. Insulin treatment was discontinued after 5 d, and on day 6, both SMGs of mice were cannulated and 5×10^9^ ppg of either rAd-Control (empty vector) or rAd-proinsulin-B10 were delivered. This dose was chosen because there was little vector dissemination via circulation and did not significantly decrease blood glucose levels in non-diabetic mice (Fig. 5c). However, this dose did achieve serum hProinsulin concentrations similar to the levels seen in the I.V. delivery experiment using 2×10^9^ vector particles (Fig. 3b).

**Figure 6:**
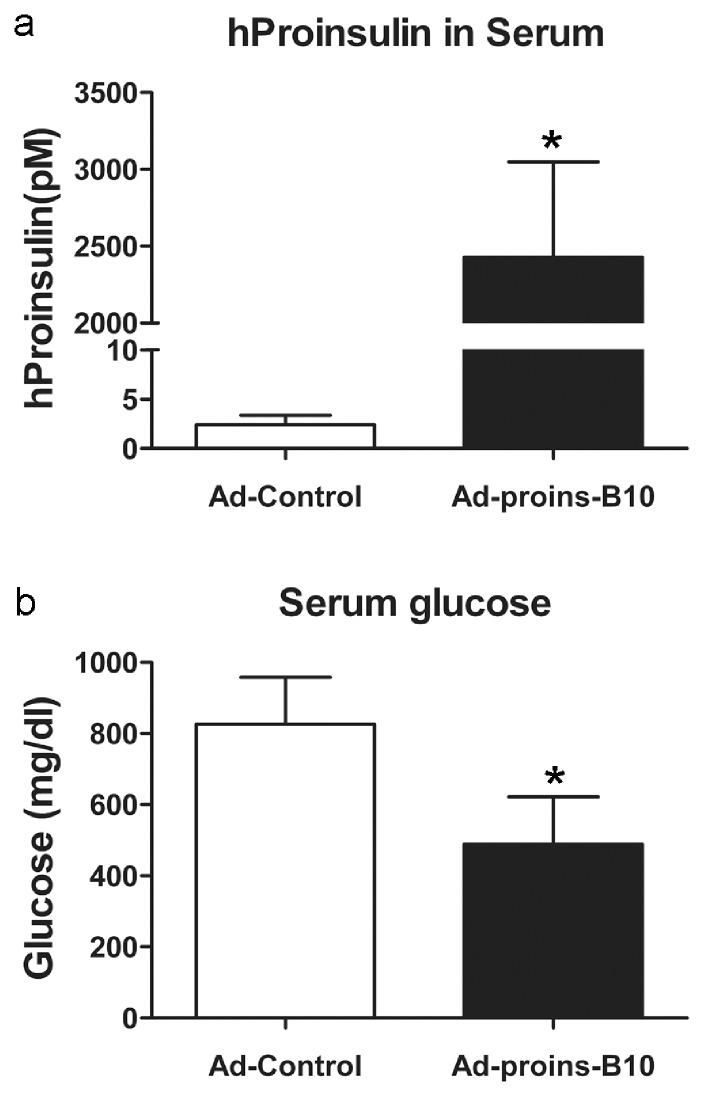
Endocrine secretion of hProinsulin from SMGs transduced with rAd-proinsulin-B10 significantly reduces blood glucose in ALX-diabetic mice. Both SMGs of ALX-diabetic mice were transduced with 5×10^9^ particles of either rAd-Control (empty vector) or rAd-proinsulin-B10. Sera were collected 24 h later. (**a**) ELISA analysis of hProinsulin (pM) in serum, *P = 0.0003 vs. rAd-Control. (**b**) Serum glucose levels, *P = 0.036 vs. rAd-Control. For all panels, bars indicate mean+SEM for n = 7 animals per treatment group. Statistical significance was determined by unpaired Student t test followed by Mann-Whitney post hoc analysis.

At 24 h post-cannulation, the diabetic mice treated with the B10 vector had serum levels of hProinsulin in the 2.5 nM range compared to <5 pM in the control group (Fig. 6a). Additionally, serum glucose levels were significantly reduced in this group of mice compared to control group (Fig. 6b).

## Discussion

Expression and secretion of mature insulin requires a dedicated cell that contains the appropriate compartments and machinery to fold and process proinsulin to its final mature form and then secrete mature insulin in a regulated manner. The pancreatic β-cell fulfils these criteria perfectly; however, in cases where the β-cell is malfunctioning, defects in insulin production and secretion give rise to a diabetic phenotype. One mode of treatment is to replace insulin by injection of the purified protein. Alternatively, production of insulin from other, non-pancreatic tissues using gene therapy approaches is emerging as an increasingly feasible treatment option [11], [12], [13], [14], [15], [16]. Selection of a target tissue for exogenous insulin gene expression is of major importance particularly since proinsulin requires a cell with the appropriate prohormone processing enzymes, prohormone convertases 1 and 2 [33], [34] and carboxypeptidase E (CPE) [35], [36]. As such, proinsulin gene expression in non-endocrine cells that do not express these enzymes would result primarily in proinsulin secretion. The question as to whether proinsulin could be used as a treatment for diabetes is therefore a pertinent one given that access to non-endocrine target organs for gene therapy is less invasive than access to endocrine tissues. We considered the use of proinsulin as a possible treatment for hyperglycemia based on the literature demonstrating that proinsulin can help regulate glucose levels [22], [23] and also based on our observations of the CPE knock out (KO) mouse [37].

CPE is an enzyme that is intricately involved in the production of insulin. The CPE KO mouse develops diabetes, obesity and is infertile due to a defect in the production of peptide hormones such as insulin, α-melanocortin stimulating hormone and gonadotropin releasing hormone, respectively [37], [38]. The CPE KO diabetic phenotype develops as glucose intolerance as early as week 12 and slowly develops to hyperglycemia at week 17, with the peak at week 30 that is level for approximately 2 weeks. Concomitant with the hyperglycemic profile, circulating proinsulin levels increase within a similar timeframe, such that fasting proinsulin levels (60–70 ng/ml, (∼5.3–6.2 nM) [37]) significantly exceed normal fasting levels of insulin (<0.5 ng/ml, (<∼86 pM)) in WT mice. Of note in the CPE KO mice was the subsequent reversal of the diabetic phenotype once the levels of proinsulin peaked at ∼32 weeks [37]. Since proinsulin has insulinotrophic properties, albeit at lower efficacy [22], [23], and studies on fat cells show that supramaximal levels of proinsulin can induce glucose uptake [37], it was reasonable to conclude that the reversal of hyperglycemia in CPE KO mice was due to the high levels of circulating proinsulin. Hence, the experimental design here focused on the use of proinsulin as a potential treatment for diabetes *in vivo*.

To reduce sorting of hProinsulin to the RSP and subsequent secretion into saliva when expressed in SG cells *in vivo*, we utilized a human proinsulin variant (hProinsulin-B10) found in Familial Hyperproinsulinemia (FH) [19], [20], [39]. FH is a human disease where defects in the proinsulin gene sequence and/or β-cell prohormone processing machinery results in elevated levels of circulating proinsulin. Patients carrying mutations in proinsulin that result in this phenotype can be asymptomatic, but are more typically prone to diabetes due to the imbalance in the insulin:proinsulin ratio [40]. It has been shown that proinsulin-B10 is poorly trafficked to the RSP in pancreatic β-cells resulting in ∼15–25% of newly synthesized proinsulin-B10 being secreted through the constitutive secretory pathway [20], [21].

Proinsulin-B10 is predicted to be more active than the WT proinsulin form, since mature insulin-B10 has a binding affinity for the InsR approximately 500% higher than WT insulin [41]. Hence, we predicted that hProinsulin-B10 expressed from SGs would be secreted into the circulation via the constitutive secretory pathway, and have sufficient biological activity to remediate a diabetic phenotype similar to that seen in the hyperproinsulinemic CPE KO mouse. Indeed, our expressed hProinsulin-B10 was bioactive as demonstrated by stimulation of InsR signaling in 293 cells (Fig. 2), although its activity was comparable to hProinsulin-WT in this assay.

After validation of our vector constructs by transduction of several cell lines (Fig. 1) and confirming the bioactivity of the expressed protein (Fig. 2), we focused on the primary goals of the project: 1) to compare the efficacy of gene transfer between three different delivery routes and 2) to test the efficacy of utilizing a human proinsulin-B10 gene to treat a diabetic phenotype in rodents. The results show that using all three delivery routes, hProinsulin-B10 was found in the circulation, demonstrating effective expression, trafficking and secretion through the constitutive pathway as predicted. However, expression and secretion from the PTG gland (Fig. 4b) was significantly lower than from the tail vein (Fig. 3b) or SMG (Fig. 5b). In all but one time point (Fig. 5a, 48 h, 1×10^10^ ppg Ad-B10), the serum glucose levels were unchanged statistically by the expression of the proinsulin. However, this was expected since normo-glycemic mice were used in these experiments and likely compensated for the activity of hProinsulin to maintain glucose homeostasis. Vector delivery via tail vein injection produced significant levels of hProinsulin likely to be from the liver; however, vector particle containment is a potentially serious problem with this route since the particles were found at high levels in several organs tested. The PTG is the preferred target gland for clinical gene transfer [4] however in this animal model, the amount of proinsulin produced from PTG was not considered sufficient and may be due to a reduced capacity for transgenic protein production (i.e. – the PTG is a small gland in mice with fewer epithelial cells) or due to the technical difficulty inherent in the PTG gland cannulation procedure in mice versus humans.

We therefore chose to use SMG delivery in the diabetes treatment experiment since it was more accurate and efficacious when compared to the PTG delivery and led to generally similar levels of circulating transgenic hProinsulin-B10 as tail vein delivery. The achieved levels of hProinsulin-B10 in the circulation (∼2–3 nM, Fig. 6a) are also similar to the levels found in the CPE KO mice [37] and were sufficiently high to have a glucose-lowering effect in diabetic animals. Although this experiment was concluded only 24 h after vector delivery, it demonstrates a proof of concept for the use of proInsulin expression and secretion from this SG to ameliorate hyperglycemia. Longer time points were not considered because the vectors used were constructed with the CMV promoter, which is prone to gene silencing by methylation soon after SG delivery [42]. Even with this short period of transgene expression, we observed significant reduction of circulating glucose within 24 h and concluded that rAd vector mediated expression of hProinsulin-B10 from the SMG attenuated hyperglycemia in diabetic mice.

A foreseeable limitation with our current expression vector is the possibility of causing hypoglycemia under conditions where chronically high levels of proinsulin are present in an unregulated manner. Also, the possible non metabolic roles of proinsulin via activation of the ERK pathway through the insulin receptor A isoform to elicit cell proliferation and migration [43] would also have to be considered in the use of proinsulin-B10. In the future, however, lower vector doses may be more desirable especially when considering the longer half life of proinsulin compared to insulin and that the levels of circulating hProinsulin-B10 detected in this experiment were higher than those observed in FH patients (278–386 pM [40]). Studies are underway to develop adeno associated virus (AAV) vectors customized with promoters that would allow regulation and titration of gene expression. Promoter regulation may avoid potential complications associated with chronically high circulating levels of proinsulin such as described for cardiovascular diseases [44], [45], [46], although a recent genome-wide study found no direct association of proinsulin and coronary arterial disease pathogenesis [47]. The studies reported here provide the foundation for potential SG-mediated treatment of diabetes using gene transfer technology. Future studies using advanced vectors and additional type II diabetes models, including a large animal model, are needed to confirm the utility of the SG as a feasible gene therapy target for treatment of this serious endocrine disease.

## Supporting Information

Figure S1 **Confluent HEK293 cells were serum starved for 2 h and then incubated in serum-free medium containing increasing amounts (0–50 nM) of recombinant human insulin (rhInsulin) for 15 min.** Protein lysates were collected and analyzed by Western blot for phospho-Akt(Ser473) and total Akt. Immunoblot band intensities were measured and bars indicate mean ± SEM of the P/T Akt ratio for n = 3 samples.
(TIF)

Methods S1 **Phosphorylation of Ser473-AKT in HEK293 cells treated with recombinant human insulin (rhInsulin).** HEK293 cells were seeded in 6-well tissue culture dishes at a density of 4×10^5^ cells/well and allowed to attach overnight. Cells were then switched to serum free medium (SFM) for 2 h to decrease surface receptor signal transduction. Two to 3 wells/group were then treated with 0–50 nM rhInsulin (Sigma) diluted in SFM. After 15 min, the cells were harvested and the cell lysates were analyzed by Western blot for phospho-AKT (Ser473) and total Akt as described in the main manuscript.
(DOCX)
