# Supplementary figures and images for: Expression and Secretion of Human Proinsulin-B10 from Mouse Salivary Glands: Implications for the Treatment of Type I Diabetes Mellitus
Source: PLoS One. 2013 Mar 15;8(3):e59222. doi: 10.1371/journal.pone.0059222 (PMC3598661; doi:10.1371/journal.pone.0059222)

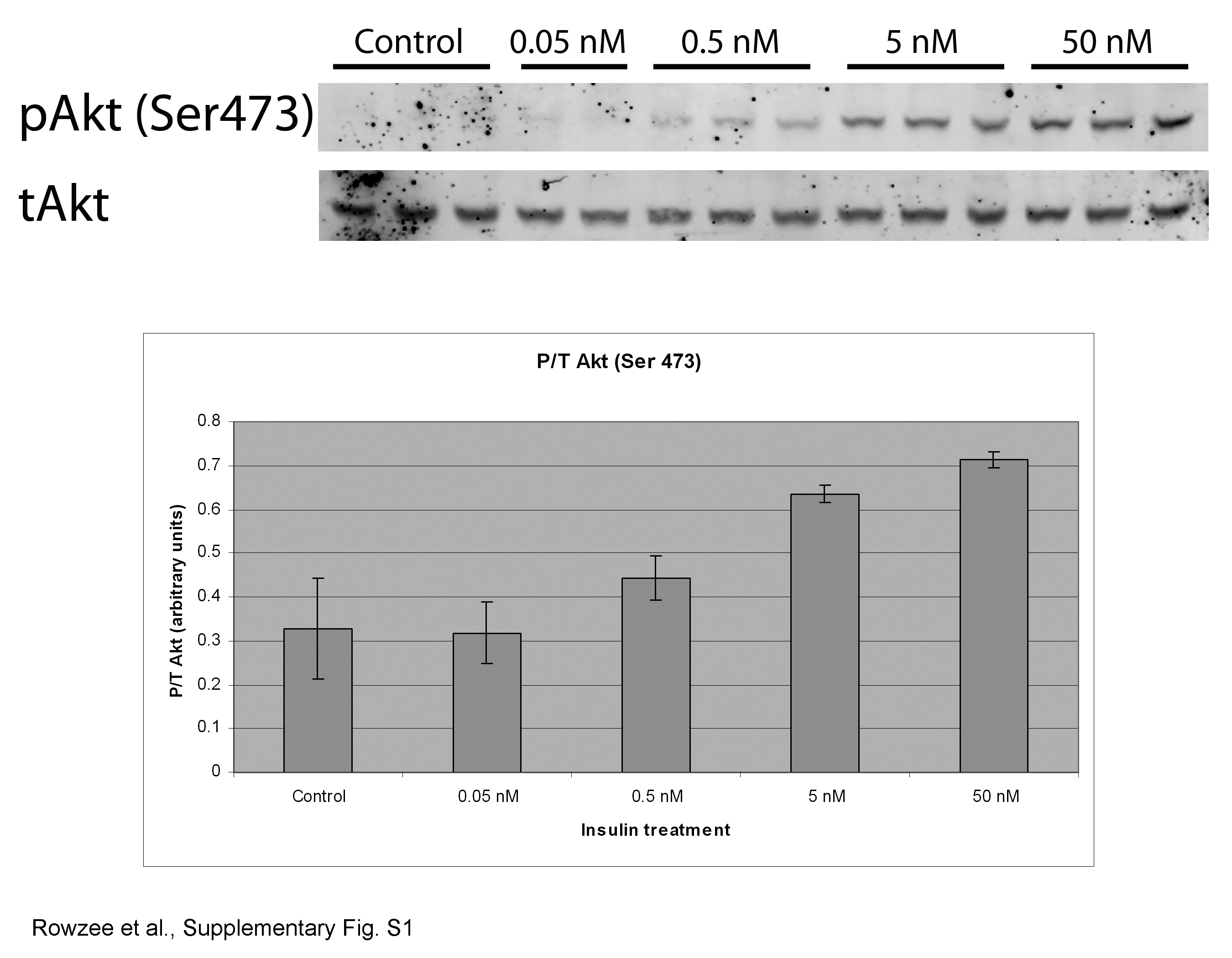

Supplement: Figure S1 — Confluent HEK293 cells were serum starved for 2 h and then incubated in serum-free medium containing increasing amounts (0–50 nM) of recombinant human insulin (rhInsulin) for 15 min. Protein lysates were collected and analyzed by Western blot for phospho-Akt(Ser473) and total Akt. Immunoblot band intensities were measured and bars indicate mean ± SEM of the P/T Akt ratio for n = 3 samples. (TIF) [file pone.0059222.s001.tif]
