# Supplementary material for: Expression and Secretion of Human Proinsulin-B10 from Mouse Salivary Glands: Implications for the Treatment of Type I Diabetes Mellitus
Source: PLoS One. 2013 Mar 15;8(3):e59222. doi: 10.1371/journal.pone.0059222 (PMC3598661; doi:10.1371/journal.pone.0059222)
Supplement: Methods S1 — Phosphorylation of Ser473-AKT in HEK293 cells treated with recombinant human insulin (rhInsulin). HEK293 cells were seeded in 6-well tissue culture dishes at a density of 4×10^5^ cells/well and allowed to attach overnight. Cells were then switched to serum free medium (SFM) for 2 h to decrease surface receptor signal transduction. Two to 3 wells/group were then treated with 0–50 nM rhInsulin (Sigma) diluted in SFM. After 15 min, the cells were harvested and the cell lysates were analyzed by Western blot for phospho-AKT (Ser473) and total Akt as described in the main manuscript. (DOCX) [file pone.0059222.s002.docx]

**Supplemental Methods**

**Phosphorylation of Ser473-AKT in HEK293 cells treated with recombinant human insulin (rhInsulin):** HEK293 cells were seeded in 6-well tissue culture dishes at a density of 4x10^5^ cells/well and allowed to attach overnight. Cells were then switched to serum free medium (SFM) for 2h to decrease surface receptor signal transduction. Two to 3 wells/group were then treated with 0-50 nM rhInsulin (Sigma) diluted in SFM. After 15 min, the cells were harvested and the cell lysates were analyzed by Western blot for phospho-AKT (Ser473) and total Akt as described in the main manuscript.
